# Wood Residue-Derived Biochar as a Low-Cost, Lubricating Filler in Poly(butylene succinate-*co*-adipate) Biocomposites

**DOI:** 10.3390/ma16020570

**Published:** 2023-01-06

**Authors:** Miriam Cappello, Damiano Rossi, Sara Filippi, Patrizia Cinelli, Maurizia Seggiani

**Affiliations:** Department of Civil and Industrial Engineering, University of Pisa, Largo Lucio Lazzarino 2, 56122 Pisa, Italy

**Keywords:** Poly(butylene succinate-*co*-adipate), PBSA, biochar, biopolymers, biocomposite, thermoplastic composites, injection moulding, lubricant, filler

## Abstract

This study focused on the development of a novel biocomposite material formed by a thermoplastic biodegradable polyester, poly(butylene succinate-*co*-adipate) (PBSA), and a carbonaceous filler as biochar (BC) derived by the pyrolysis of woody biomass waste. Composites with various BC contents (5, 10, 15, and 20 wt.%) were obtained by melt extrusion and investigated in terms of their processability, thermal, rheological, and mechanical properties. In all the composites, BC lowered melt viscosity, behaving as a lubricant, and enhancing composite extrudability and injection moulding at high temperatures up to 20 wt.% of biochar. While the use of biochar did not significantly change composite thermal stability, it increased its stiffness (Young modulus). Differential scanning calorimeter (DSC) revealed the presence of a second crystal phase induced by the filler addition. Furthermore, results suggest that biochar may form a particle network that hinders polymer chain disentanglement, reducing polymer flexibility. A biochar content of 10 wt.% was selected as the best trade-off concentration to improve the composite processability and cost competitiveness without compromising excessively the tensile properties. The findings support the use of biochar as a sustainable renewable filler and pigment for PBSA. Biochar is a suitable candidate to replace more traditional carbon black pigments for the production of biodegradable and inexpensive innovative PBSA composites with potential fertilizing properties to be used in agricultural applications.

## 1. Introduction

The increasing interest in the development and optimization of new sustainable materials is driving research in the direction of alternative biodegradable polymers to traditional petroleum-based plastics. Among these, poly(butylene succinate-*co*-adipate) (PBSA) is one the most popular biodegradable polymer with excellent mechanical properties, and processability via extrusion, film blowing, thermoforming, and injection moulding similar to polyolefins [1,2,3]. PBSA is a thermoplastic aliphatic random co-polyester obtained by direct esterification of succinic acid and adipic acid with 1,4-butanediol. Commercially, PBSA is offered in a variety of grades with molecular weights ranging from 10^5^ to 10^6^ g/mol. Despite typically being synthesized from fossil fuels, monomers of PBSA can also be manufactured from bio-based feedstock. Some manufacturers produce partially bio-based poly(butylene succinate), PBS, and PBSA with a high bio-based content (up to 54 wt.%) derived from bio-based succinic acid [4]. PBSA exhibits enhanced properties than PBS: the lower crystallinity and higher flexibility of PBSA polymer chains result in reduced tensile strength and melting point, higher impact strength, and greater elongation at break [5,6]. In addition, PBSA presents a higher degradation rate in industrial composting, soil, seawater, and activated sludges [2,4,7,8]. For these reasons, PBSA is more attractive than PBS in all the applications where biodegradability in soil and water is a key factor. In this context, PBSA has been proposed to produce agricultural mulching films, bag liners, and plant pots [9]. Despite its promising mechanical properties and biodegradability, the spread of bio-based PBSA on the plastic market is mainly hindered by its relatively high cost (5–6 EUR/kg) [10]. For this reason, other bioplastics, such as poly-lactic acid (PLA), and more traditional petroleum-based non-biodegradable plastics are still dominating the potential PBSA market shares. A strategy to turn PBSA into a more affordable and attractive polymer for commodities consists of the addition of low-cost biodegradable and renewable fillers, producing PBSA composites with a higher market value and competitiveness. Following this approach, 30 wt.% of corn starch was blended with PBSA to make PBSA-based films with improved properties [1]. Recently, short hemp fibres (30 wt.%) were added as a natural reinforcement to create PBSA composites with the highest levels of environmental efficiency, stiffness, and tensile modulus. [11,12]. Moreover, 20 wt.% of hydrolysed collagen was mixed with PBSA to improve its processability and confer fertilizing properties to the final product [13].

Among the several fillers, carbon black is by far the dominant filler and pigment employed with natural and synthetic rubbers in automotive tires, mechanical rubbers, and elastomeric polymers. Carbon black is a para-crystalline fine powder of different sizes, porosity, and shapes that is formed as the result of the partial combustion of hydrocarbons such as coal and tar. It can be mixed with polypropylene to absorb ultraviolet radiation, thus lowering material degradation, in laser printer toners and inks, as well as in paint, coating, plastic compounding, and electronic applications [14,15]. Unfortunately, the several advantages offered by carbon black as a low-cost and easily accessible filler are compensated by important drawbacks. Carbon black is a product derived from non-renewable resources, such as natural gas or petroleum-based heavy oils. The formed nano-sized soot is possibly carcinogenic at low concentrations and produces respiratory discomfort at high concentrations. This problem normally occurs during particle handling and composite manufacturing, but also once waste composites are incinerated for final disposal [16,17]. Eventually, it is important to highlight that the carbon black industry is positioned within the fossil-fuel industry which is a major contributor to CO_2_ emissions implicated in global warming.

The serious environmental and health risk factors associated with carbon black production, composites manufacturing, and disposal have recently pushed the industry towards alternative “green” solutions to petroleum-based fillers. Among the various renewable sources, biomass waste, with a global production of 140 Gt per year [18], has attracted interest from researchers as a potential biosolid that can be converted through pyrolysis into biochar (BC), a useful highly surface-active, stable, porous, and functional group-rich charred material. Biochar is chemically similar to carbon black which essentially contains carbon as the main element. Biochar composition, H/C, and O/C ratios highly depend on the type of biomass used and thermal degradation. However, fixed carbon (50–90 wt.%), volatile matter (0–40 wt.%), moisture (1–15 wt.%), and ash (0.5–5.0 wt.%) are generally regarded as major constituents. High carbon content (up to 98 wt.%) and strongly aromatic structure are constant features. Biochar is hygroscopic, showing pH values in the 3–12 range, and micro and nano porosity with BET surface area from 50 to 500 m^2^/g [19,20], depending on the biomass used and production process.

Although BC is frequently used for water pollutant cleanup and soil amendment, its application outside these sectors is an emerging strategic field in the material science industry [19,21,22]. In particular, the area of polymer composite manufacturing using BC fillers is a growing area of research and represents the subject of the current work. Das et al., 2015 conducted an initial groundbreaking investigation on the usage of BC in polymer composites. The authors demonstrated the enhanced mechanical properties of wood-polypropylene matrices by the addition of 24 wt.% BC filler [23]. Since then, BC fillers have been widely studied in different thermoplastic formulations, such as polyolefins, ultra-high-density polyethene, polyamide, polyesters (PLA, PBT, PTT, and PET), and polycarbonates [24,25,26,27]. Due to their numerous applications, BC-epoxy resins are one of the most researched thermosetting matrices. The unsaturated polyester resins that are mostly used as building materials make up the other major class of thermosetting BC-based composites. Recently, BC has been successfully composted with nano-silica as filler for PBS rubbers [28].

In this context, the present research work aims to fully exploit the advantages of a bio-based carbon filler, such as biochar, with a bioplastic as PBSA in the creation of inexpensive, high-performance composites that have improved characteristics over neat PBSA. To our knowledge, only petroleum-based carbon fillers, such as carbon black, have been investigated in PBS composites by Ge et al. 2017 [29]. The authors studied the non-isothermal crystallization of 3 wt.% PBS/carbon black composites and reported good dispersion of carbon black aggregates in the PBS matrix. The composites showed higher mechanical elastic modulus (up to 20%) and electrical conductivity from 10^−12^ to 10^−6^ S/cm than virgin PBS. Employing biochar in PBSA bio-composites could have multiple advantages; first, it would provide more effective utilization of a waste which otherwise would be landfilled; second, it could lower the overall cost of the PBSA raw material also offering an alternative pigmenting solution to traditional carbon black fillers; lastly, biochar efficiency as a soil amendment and organic contaminants cleanup agent could open up opportunities for the development of fully biodegradable and sustainable innovative products within the agricultural sector.

The use of biochar filler in PBSA was evaluated by producing via melt extrusion composite pellets with 5, 10, 15, and 20 wt.% of BC. The produced composites were subjected to scanning electron microscopy (SEM) and Fourier Transform Infrared (FTIR) analysis to assess the dispersibility of the BC into the polymer matrix and to ascertain the molecular interactions between PBSA and BC, respectively. Differential Scanning Calorimetry (DSC) and Thermal Gravimetric Analysis (TGA) were used to determine the thermal behaviour of PBSA/BC composites. To assess the impact of BC on melt viscosity and processability, the rheological behaviour of the composites was also examined. Finally, the evaluation of the mechanical properties was determined by tensile tests conducted on moulded dog-bone specimens.

## 2. Experimental

### 2.1. Materials

Compostable and food contact certified (EU10/2011) PBSA pellets (BioPBS^TM^ FD92PM) were purchased from Mitsubishi Chemical Co. (MCPP), Tokyo, Japan. The PBSA polymer is appropriate for blown film applications, has a melting temperature of 84 °C, a density of 1.24 g/cm^3^, and a Melt Flow Rate (MFR) (ISO 1133, 190 °C, 2.16 kg) of 4 g/10 min.

Biochar of high class I quality (Italian Decree 75/2010) obtained by pyro-gasification of woody biomass was supplied by BioDea srl (Arezzo, Italy) in form of micro-size powder “black silt”. This biochar filler is a stable porous soil improver containing 3–5 wt.% of ash, 67–75 wt.% of carbon, 0.2–0.4 wt.% of total N, 0.03–0.04 wt.% P, and traces of elements as Fe, Na, K, Ca, and Mg. The particles as received were sieved to obtain a final particulate in the 5–60 µm size range for the subsequent processing and characterizations.

### 2.2. Composite Production

Initially, the biochar powder was dried at 105 °C for 24 h to remove moisture (typically 5–6 wt.%) and the virgin PBSA pellets were dried at 55 °C for 24 h. Then, PBSA/BC mixtures with 5, 10, 15, and 20 wt.% BC (indicated as PBSA5, PBSA10, PBSA15, and PBSA20, respectively), were processed by a single-screw Brabender Extruder GmbH & Co. KG. Table 1 lists the temperature settings for the extruder for all the composites (from the first zone following the main feeder to the head zone). The screw was rotated at a constant 60 rpm (23 Nm of torque). The extruded filaments (approximately 2 kg/h) were chilled in a water bath at room temperature and reduced in granules by an automatic pelletizer. The pellets were subjected to thermal, rheological, morphological, and mechanical characterizations after being dried for roughly 24 h at 55 °C in an oven. To check whether the composite pellets were completely dry, a given amount of composite granules was weighed over time until a stable and constant value was reached. Finally, the composite granules were vacuum-sealed to prevent moisture absorption during their storage.

### 2.3. Characterization

#### 2.3.1. Thermal Analysis

PBSA and BC starting materials as well as PBSA/BC composites were subjected to thermogravimetric analysis (TGA) using a NETZSCH STA 2500 Regulus (GmbH, Selb, Germany). Each sample was added to a platinum pan in amounts of about 15 mg, and it was heated under a nitrogen atmosphere from room temperature to 800 °C at a rate of 10 °C/min. TGA was employed to assess the thermal stability of the raw materials and composites, and their processing via hot melt extrusion.

Differential scanning calorimetry (DSC) was conducted on BC, PBSA, and PBSA/BC composites to evaluate the effect of BC content on PBSA crystallinity using a Perkin Elmer Instrument (Pyris 1 DSC 6000, Waltham, MA, USA) under 50 mL/min nitrogen flow. About 20 mg of each sample were placed in a hermetically sealed aluminium pan and initially heated at a rate of 10 °C/min from −50 °C to 120 °C to erase any thermal history from processing. The sample was maintained at 120 °C for 3 min, subsequently cooled down to −50 °C at 10 °C/min, and finally heated up to 120 °C at 10 °C/min. The second heating step measurements of the melting temperature (T_m_) and enthalpy (∆H_m_) were utilized to determine the degree of crystallinity (X_c_) of PBSA composites using Equation (1):

(1) $$X_{c}\left(\%\right)=\frac{{\Delta H}_{m}}{\Delta H_{m}^{0}\cdot f_{w}}\cdot 100$$

where ΔH^0^_m_ is the melting enthalpy per gram of the PBSA polymer that is 100 % crystalline (142 J/g [30]), and f_w_ is the weight fraction of PBSA in the composite. The DSC curves reported in the article refer to the second heating cycle.

#### 2.3.2. Rheological and Mechanical Analysis

Melt rheological measurements were performed at 160 °C and 170 °C, using an MCR 92 Rheometer from Anton Paar in Italy with a plate-plate geometry (25 mm diameter, 1 mm gap). All samples were dried before testing. To erase any processing-related thermal history, each sample pellet was melted on the rheometer plate at constant temperature for 3 min. An amplitude sweep was used to select the operating parameters to find the linear viscoelastic limit. Using a strain of 0.2 %, tests were run with oscillatory frequency sweeps from 0.05 to 100 Hz (0.314 to 628 rad/s). The complex viscosity η*, the loss modulus G″, and the storage modulus G′ were all measured as functions of angular frequency ω.

The analysis of flow behaviour was carried out by Melt Flow Rate (MFR) measurements following UNI EN ISO 1133 using a Benedetto Campana, Milano, Italy instrument. A steady load of 2.16 kg was applied to the barrel that was heated to 150 °C and used to extrude about 4 g of pellets through the normalized diameter (2.095 mm). At least three replicates were performed for each sample, and the standard deviations for each data point were recorded.

Tensile tests were performed on dog-bone specimens of PBSA, and PBSA/BC composites were produced using a mini-injection press (ZWP Proma, Poland). To produce the dog-bone specimens, the pellets were loaded in the thermostatic barrel of the injection press at 140 °C. After roughly 1 min the melt was mechanically injected within the stainless steel dog-bone mould and maintained at 60 °C for another extra minute before removing the sample. The dimensions of the dog-bone specimens were: large section width = 12 mm, narrow section width = 4 mm, thickness = 2 mm, and length = 80 mm. An Eden Prairie, MN, USA, MTS 50 KN system machine, outfitted with a 2 kN load cell and connected to a PC running the dedicated MTS Elite Software TW, was used to conduct stress–strain testing at room temperature at a crosshead speed of 10 mm/min. Each tensile test was performed on five specimens and the standard deviation for each data point was reported.

#### 2.3.3. Morphological Analysis

PBSA and PBSA/BC pellets were fractured in liquid nitrogen. Scanning electron microscopy (SEM) COXEM Co., Ltd., Daejeo, Korea, Model EM-30N was used to examine the fractured surfaces. To induce electroconductivity, the sample fractured surfaces were coated before SEM analysis with a uniform layer of Pt (5–6 nm thickness) using an SEM coating apparatus (Edward Spotter Coater). Energy Dispersive Spectroscopy (EDS) was carried out on pellets of biochar and PBSA composite using an FEI Quanta 450 FEG ESEM instrument (Hillsboro, OR, USA).

#### 2.3.4. Fourier Transform Infrared Analysis

FTIR spectra were acquired to account for the possible molecular interactions between the functional groups of PBSA and those of biochar. The characteristic absorption peaks of the two components were tracked using FTIR spectroscopy in specified areas. The FTIR spectra were captured using a Perkin Elmer Spectrum 400 FT-IR Spectrometer (Perkin Elmer, Waltham, MA, USA), outfitted with a Perkin Elmer Universal ATR Sampling Accessory, in the wavenumber range between 4000 and 650 cm^−1^ at 4 cm^−1^ scanning resolution. FTIR analysis was carried out on the pellets of PBSA, PBSA/BC composites and the BC powder; all the samples were pre-dried at 40 °C in a vacuum oven for about 24 h before analysis.

## 3. Results and Discussion

### 3.1. Thermal Properties

**Figure 1:**
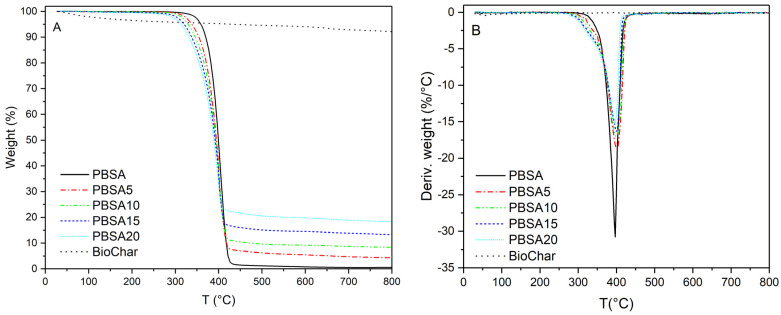
TG (**A**) and DTG (**B**) curves of BC, PBSA, and PBSA/BC composites with different BC content in nitrogen at a heating rate of 10 °C/min.

Figure 1 reports the thermogravimetric (TG) and differential thermogravimetric (DTG) curves of BC, PBSA, and PBSA/BC composites. As shown, BC presents a relatively stable behaviour with less than 10% mass loss registered between room temperature and 800 °C. This was expected since biochar is produced by a high-temperature pyro-gasification process and has been already subjected to thermal degradation. The observed BC thermograms are typical of a carbonaceous pyrolyzed lignocellulose material derived from agricultural and forestry wastes [31]. The slow mass decay is mainly ascribed to refractory organic matter and the recalcitrant nature of aromatic rings (e.g., degraded lignin) and strong C-C covalent bonds [23,32].

The PBSA thermogram shows thermal stability up to about 300 °C with a single sharp stage of thermal degradation resulting in a peak close to 400 °C. A negligible residue (0.4 wt.%) was measured at 800 °C.

The thermograms of the PBSA composites show constant and sharp mass decays which indicate a uniform dispersion of the BC filler within the polymer matrix at the various concentrations. If the biochar powder was not homogeneously dispersed within the PBSA polymer matrix, we would likely end up with individual small TGA pellets (15 mg) having a large variability of biochar content. This sample variability would lead to TG charts that are not reproducible for the same biochar concentration. Moreover, no clear trends could be observed when comparing different TG curves obtained with different biochar concentrations (PBSA5, PBSA10, PBSA15, and PBSA20). In other words, the small composite amounts used in the TGA are representative homogeneous samples of the entire macroscopic composite structure. The good dispersion of biochar is confirmed by the second temperature onsets that nicely match the PBSA/BC concentrations and follow the slight slope of BC degradation. The addition of the BC filler did not affect the degradation peaks of the composites that are positioned within the narrow temperature range between 395 °C and 400 °C. The presence of the filler led to a small shift of the first temperature onset (from 365 °C to 325 °C). This can be attributed to the initial degradation of the filler at low temperatures, a phenomenon that was also observed for collagen-based PBSA matrices [13]. As reported by Bo et al., 2022, the shift of the degradation onsets could be also associated with the faster molecular fragmentation caused by the presence of inorganic fillers when these are mixed with PBSA matrices at relatively high concentrations (>5 wt.%) [33]. Despite the small onset shift, the addition of the biodegradable BC filler did not alter significantly composite thermal resistance, thus showing the potential for BC to be employed as a renewable inexpensive bio-filler for novel PBSA composite materials. Moreover, the degradation shift did not fall within the range of PBSA melt extrusion and processability. This shows PBSA’s suitability to be processed without incurring thermal degradation.

**Figure 2:**
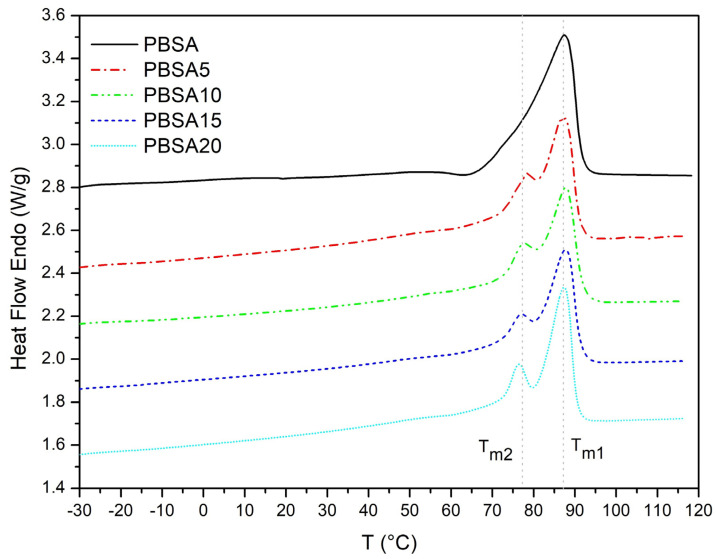
DSC curves of PBSA and PBSA/BC composites during the 2nd heating run at 10 °C/min.

Figure 2 shows the DSC curves of PBSA and PBSA/BC composites obtained in the 2nd heating run at 10 °C/min.

**Figure 3:**
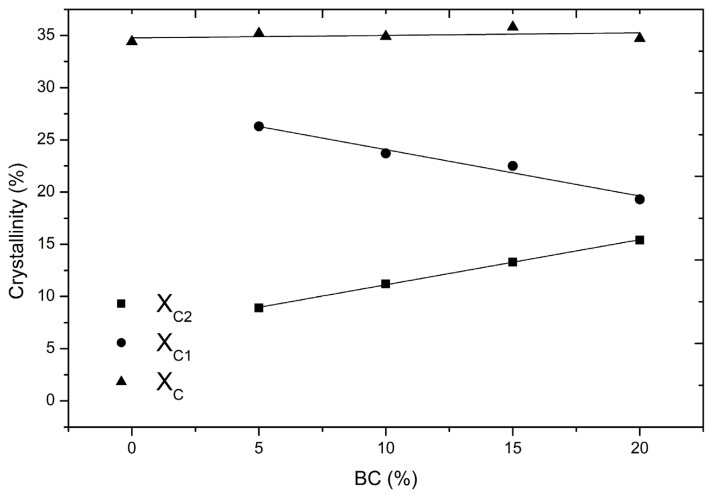
Crystallinity degree trend of PBSA and PBSA/BC composites.

The degree of crystallinity (X_C_) of PBSA and PBSA/BC composites determined by Equation (1) remains almost constant with the increase in BC content (from 34.4 to 35.8%, Table 2 and Figure 3). These crystallinity values align with those reported in similar studies using the same type of PBSA [13,34]. Although the addition of BC did not affect the total crystallinity X_C,_ this value is calculated as a sum of two endothermic peaks: a small shoulder peak at about 76–78 °C (T_m2_), followed by a large peak at about 87–88 °C (T_m1_). The shoulder peak is not present in the pure PBSA and only appears in the PBSA composites. PBSA/BC composites maintain the same level of total crystallinity (X_C_) equal to the pure polymer; however, the proportion of crystallinity estimated by the areas under the peaks associated with the two endothermic events changes slightly with BC concentration; in particular, the higher the BC content, the greater is the crystallinity associated with the shoulder peak (X_C2_), and, in turn, the smaller the crystallinity associated with the large peak (X_C1_).

PBS and PBSA heating curves obtained under non-isothermal conditions generally present one main melting event. However, they can also exhibit cold crystallization peaks followed by additional melting events associated with different populations of crystals having different thermal stability depending on the specific temperature profiles, molecular weights, strain, and presence of impurities and fillers [34]. These multiple peaks normally appear during rapid cooling that leads to the formation of different crystal phases (generally named α and β forms) that further melt as soon as the temperature increases [35,36]. The presence of a filler such as biochar can lower the nucleation energy barrier of one crystal phase over the other one, thus triggering the formation of these two crystal phases selectively: one that melts at a lower temperature (T_m2_) and the other one that melts at higher temperature (T_m1_) [37]. The linear trend between X_C2_ and PBSA/BC concentration (Figure 3) is probably associated with the increasing content of biochar particles that provide heterogeneous sites for the crystallization and melting of one specific crystal form. Despite the complex PBSA, crystallinity behaviour is still debated and the full understanding of PBSA-filler interactions is beyond the scope of the current work, our results confirm previous findings showing that organic biochar fillers act mainly as nucleating agents in elastomer composites. For instance, biochar can initiate the crystallization of polypropylene (PP) [38,39], or induce the formation of intermediate crystal phases in polyethene terephthalate (PET) composites [40]. The fact that, in our case, both the positions of the crystal peaks (T_m1_ and T_m2_) remain unmodified with the addition of BC, suggests that no relevant chemical interactions occur between the polymer and the filler which mainly acts as a heterogeneous active agent promoting PBSA crystal nucleation and growth.

### 3.2. Fourier Transform Infrared Analysis

**Figure 4:**
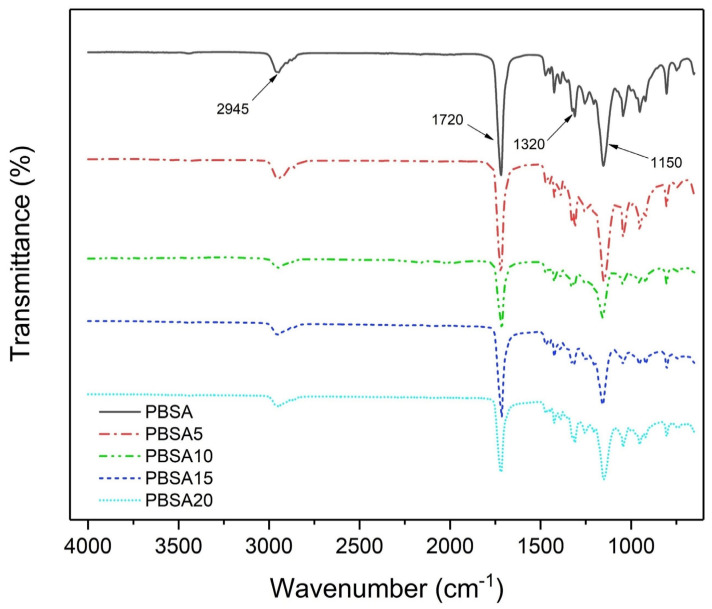
FTIR spectrum of neat PBSA and PBSA/BC composites.

The FTIR spectra of PBSA and PBSA/BC composites reported in Figure 4 confirm the non-specific molecular interactions between PBSA and biochar. The spectrum of the virgin PBSA matches with the PBSA/BC composites spectra and no relevant chemical shift and/or appearance of other bands associated with filler-matrix bonds are observed comparing the various spectra. It is important to note that biochar is a carbon-based compound with no relevant FTIR absorption bands displayed in the 4000–650 cm^−1^ wavenumber range. In light of this, the peaks at 1320 cm^−1^ and 2945 cm^−1^ were attributed, respectively, to the symmetric and asymmetric vibrations of CH_2_ groups in the PBSA main chains. The primary band at 1720 cm^−1^ was ascribed to the C = O stretching vibrations of PBSA ester groups, while the band at 1150 cm^−1^ was caused by the stretching of the -C-O-C- group in the ester linkages of PBSA [41]. In conclusion, biochar acts as pure filler damping the electromagnetic IR signal. As a result, the various PBSA peaks result in less sharpness as biochar content increases.

### 3.3. Rheological and Mechanical Properties

**Figure 5:**
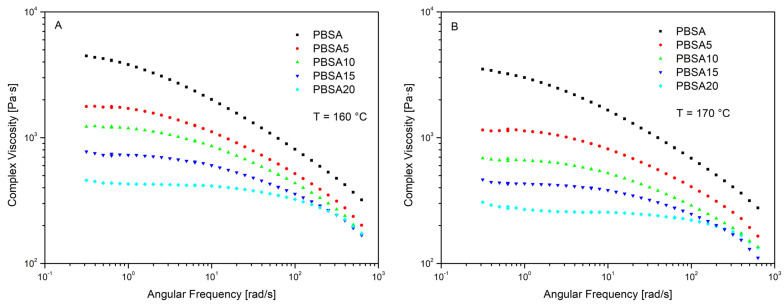
Complex viscosity versus angular frequency of PBSA and PBSA/BC composites at 160 °C (**A**) and 170 °C (**B**).

The complex viscosity versus angular frequency is reported in Figure 5 for the virgin PBSA and PBSA/BC composites at 160 °C and 170 °C. As expected for this type of polymer composite, the trends show the typical rheological behaviour of a non-Newtonian fluid with a complex viscosity that decreases as the angular frequency and temperature increase. This results from the disentanglement of PBSA polymer chains, a phenomenon which indicates a shear thinning flow behaviour of both PBSA and PBSA/BC composites [42]. The addition of the biochar filler lowers the viscosity of the composites compared to the neat PBSA, therefore increasing its processability and extrudability. As a result, the higher the filler concentration, the lower the measured complex viscosity. This behaviour can be attributed to the poor chemical interaction at the polymer-filler interface and the lubricant effect offered by the partial degradation of the biochar lignocellulose material to low molecular weight fractions [26]. As the biochar concentration increases, η* decreases more gradually till approaching a quasi-Newtonian η* plateau within the low–mid frequency range for both PBSA15 and PBSA20 composites. This may be due to the presence of the filler particles dispersed in the matrix which hinder polymer chain disentanglement and alignment along the direction of the applied shear force. This effect becomes more relevant at high biochar concentrations where the filler has probably reached the volumetric saturation within the polymer matrix; therefore, filler particles come into contact, sticking together, and forming a network that weakens the overall composites structures [43].

**Figure 6:**
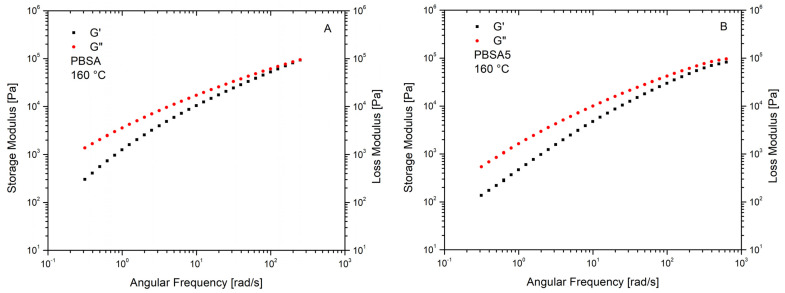

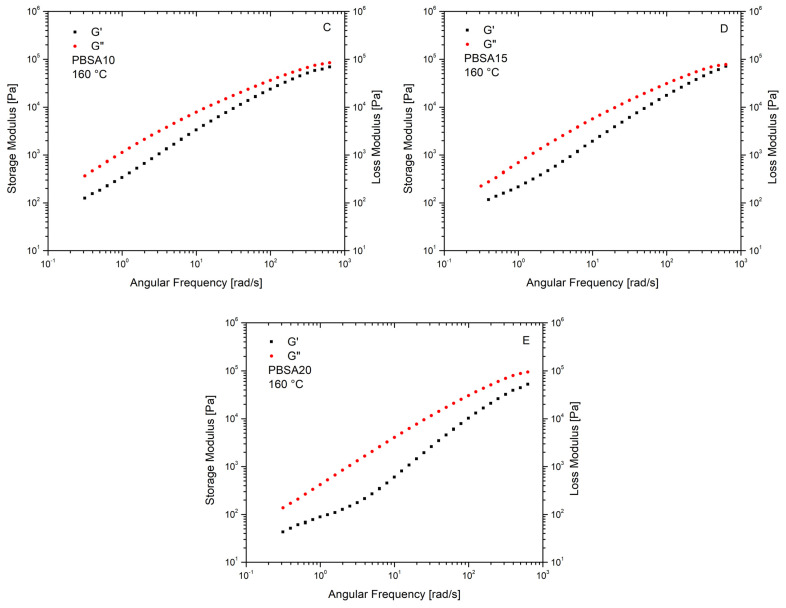
Storage G′ (**■**) and Loss G″ (**•**) moduli vs. frequency of PBSA and PBSA/BC composites at 160 °C. PBSA (**A**), PBSA5 (**B**), PBSA10 (**C**), PBSA15 (**D**), PBSA20 (**E**).

The lubricant effect of the biochar is confirmed by Figure 6 which shows the storage and loss moduli of both neat PBSA and PBSA/BC composites measured at 160 °C. Both the loss modulus G″ and the elastic modulus G′ curves decrease with the increase in biochar concentration as a result of a decreasing composite complex viscosity. At all the experienced frequencies the various composites present a viscous component G″ which is constantly higher than the elastic component G′, and a G″–G′ gap which tends to increase with biochar concentration.

The lubricant effect of biochar in polymer matrices has been investigated by a few authors. As for carbon nanotubes, graphene, and carbon black fillers, the lubricant effect is mainly attributed to the sliding of individual graphene-polymer planes under the applied shear forces [44]. In this regard, Richard et al., 2017, studied the tribological behaviour of biochar particle dimension on BC-based unsaturated polyester resins. The authors demonstrated that increasing BC loading and decreasing particle size distribution lowered the specific wear rate and friction coefficient of the composite [45]. Similar results were achieved in the following studies which demonstrated the plasticizing effect of nano-sized red mud powder obtained during the extraction of pure alumina from Bauxite employed as reinforcement in polyester resins [46]. Despite these research studies, it is difficult to establish a connection between the lubricating properties of biochar and those of its polymer composites, primarily because of the high degree of biochar variability. In fact, the vastly different origins of biomass sources combined with the extensive operating conditions that can be used during the manufacturing processes, results in the production of biochar with heterogeneous properties, which affect the performance homogeneity of the final composites [47].

**Figure 7:**
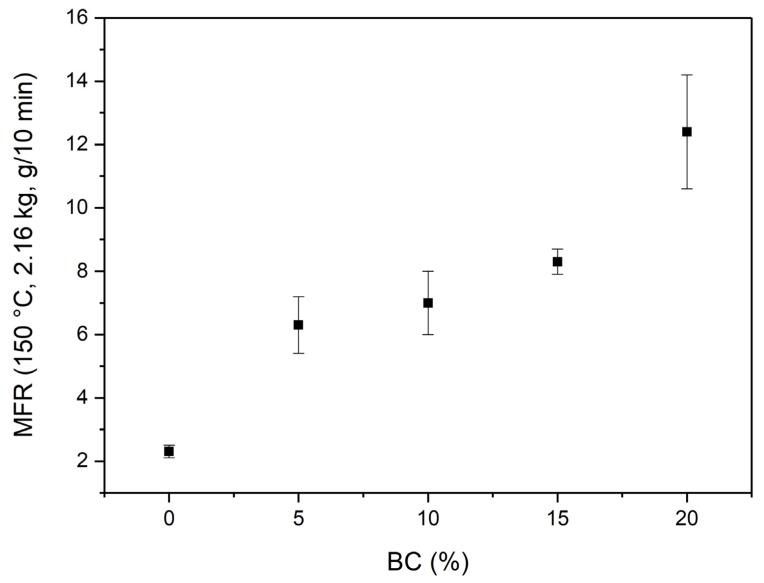
MFR at 150 °C for neat PBSA and PBSA/BC composites. Mean values ± standard deviations of 3 replicates.

The use of biochar as lubricant filler for PBSA is further verified by melt fluidity measurements (Melt Flow Rate) conducted at 150 °C on the neat PBSA and PBSA/BC composites. Results reported in Figure 7 show a linear increasing trend between the MFR and the concentration of biochar filler dispersed in the polymer matrix which increases from MFR = 2.3 ± 0.2 g/min up to MFR = 12.4 ± 1.8 g/min.

**Figure 8:**
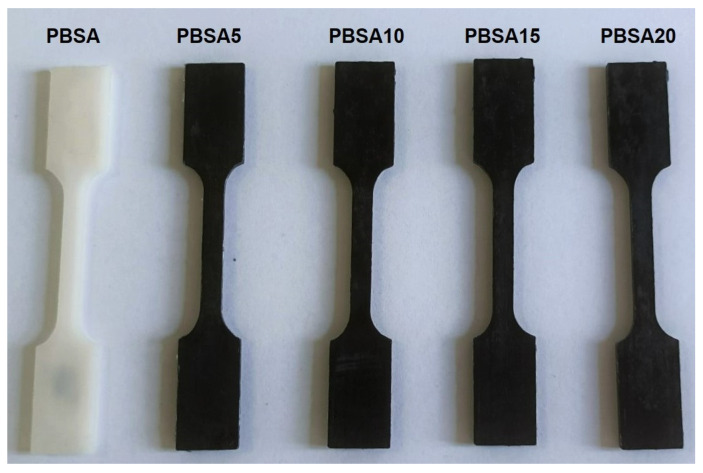
Dog-bone specimens of PBSA and PBSA/BC composites for tensile tests.

To establish the mechanical performance offered by the biochar filler at normal working temperatures, tensile tests on dog-bone specimens (Figure 8) were carried out on the various PBSA/BC composites. As shown in Table 3, the use of biochar increases Young modulus, particularly at the highest BC contents (20 wt.%), where the modulus of elasticity is about 2.5-fold increased compared to the virgin PBSA (0.29 versus 0.71 GPa). In general, the addition of biochar leads to a more rigid composite material at the expense of a lower elongation and stress at break. The stiffness effect produced by the biochar addition is relevant at high BC content (>10 wt.%) where the elongation at break drops significantly leading to a much more fragile and brittle composite material than the neat PBSA. In particular, PBSA20 showed no plasticity elongation with the yield strength and elongation at yield matching with the stress at break and elongation at break, respectively. These results are in accordance with previous outcomes by Das et al., 2015, and Poulose et al., 2018, who suggested that the presence of the biochar network and the low polymer-particle adhesion could contribute to the decrease in tensile strength and elongation of the polymer [23,38]. These findings validate our previous rheological hypothesis that explained the viscosity independence for BC content greater than 10 wt.% at low–mid frequency sweeps as a consequence of a biochar particle-to-particle network that interferes with polymer chain disentanglement. This is the same particle network which increases material stiffness in stress–strain tensile tests when BC concentration is greater than 10 wt.%. Therefore, 10 wt.% was selected as the highest BC content at which the beneficial effect offered by the biochar addition in terms of enhancement of elastic modulus (from 0.29 to 0.53 GPa), while keeping a still relatively high level of the stress at break (from 21.0 to 18.0 MPa), is not too compromised by the reduction in the elongation at break (from 400 to 188%). Moreover, the PBSA10 composite maintains the same level of yield strength and slightly lower elongation at the yield of the virgin PBSA.

### 3.4. Morphological Tests

**Figure 9:**
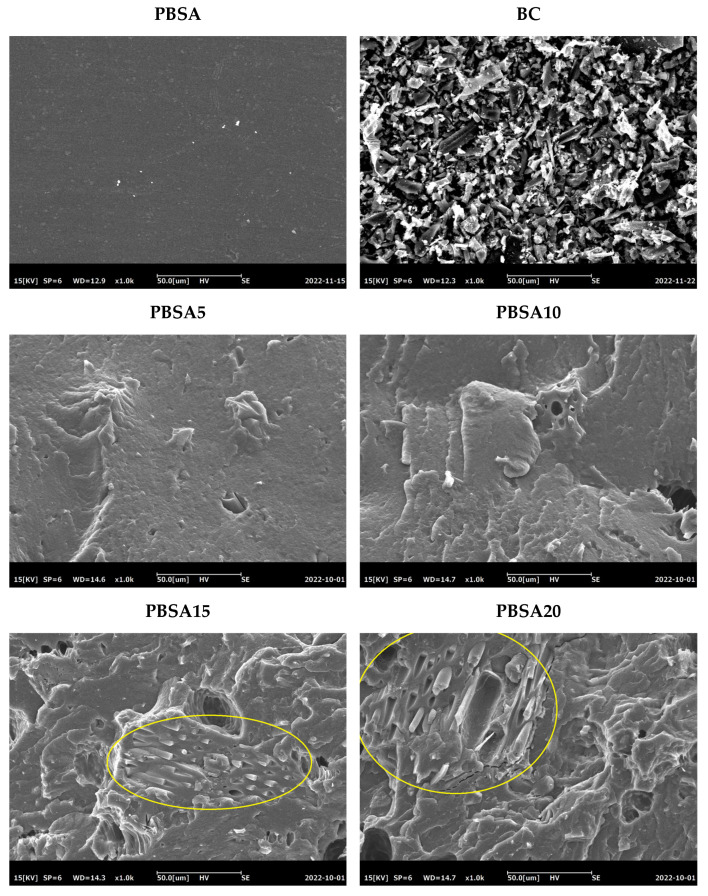
SEM images of PBSA, BC, and PBSA/BC pellets. Oriented particle streaks are highlighted in circle.

Figure 9 reports the SEM images of the freeze-fractured transversal sections of PBSA and PBSA/BC composites. The PBSA polymer surface appears compact and smooth without voids or fractures. Biochar particles have the typical morphology of a pyrolyzed milled lignocellulose particulate with an irregular flat-shaped structure. Their size was estimated via ImageJ software within the 5–60 µm range. Biochar particles are uniformly dispersed in the polymer matrices if their content remains below 10 wt.% (PBSA5 and PBSA10), whereas they form agglomerates at higher concentrations (PBSA15 and PBSA20) that are visible as oriented particle streaks on the fractured pellet surfaces. These agglomerates are the particle-to-particle networks previously discussed that may be responsible for the loss of elastic property at high biochar content, hence making the material more fragile. The various composites present some voids and cracks that could be caused by the slow biochar degradation that occurred during processing and dog-bone preparation. This level of microporosity, which is not present in the neat PBSA, could be also caused by the simple filler-matrix mechanical debonding occurring at the pellet surface during cryogenic surface fracturing.

As shown in Figure 8, PBSA/BC composites appear all equally black and uniformly pigmented with smooth clean surfaces regardless of the content of the fillers introduced to the virgin PBSA matrix. This qualitative observation opens the route to the potential use of biochar as pigmenting filler to be employed in PBSA composites as a substitute for more traditional fuel-based pigments such as carbon black [29]. In this way, a 10 wt.% biochar filler content could be also employed to reduce polymer material cost as compared to traditional carbon black which is typically added up to 3 wt.% [29]. Considering an average cost of 5–6 EUR/kg for PBSA [10], 1 EUR/kg for biochar [48], and 2 EUR/kg for carbon black [49], we estimated a reduction of about 10–11% in the cost of the PBSA/BC raw materials compared to PBSA/carbon black ones. In this regard, the use of biochar as a novel cheap pigmenting filler for biocomposite new materials such as blown films has been demonstrated in a recent publication with PBAT polymer [50].

**Figure 10:**
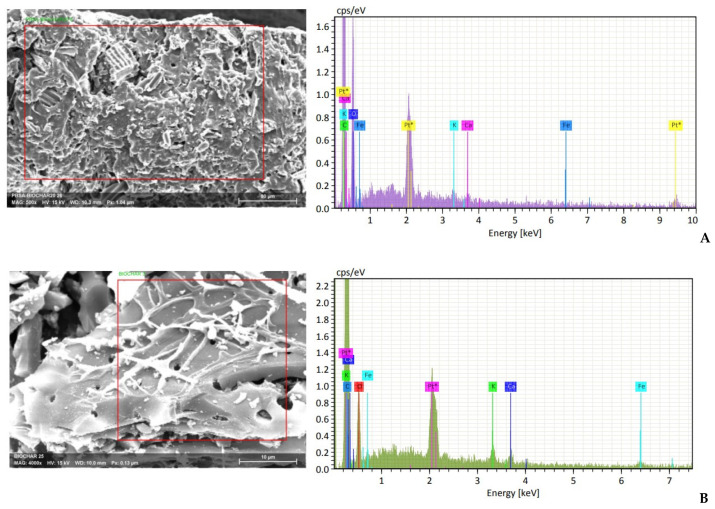
EDS microanalysis of specific areas on PBSA20 (**A**) and Biochar (**B**). The platinum Pt peak is associated with the sample surface coating.

To evaluate biochar dispersion within the PBSA matrix, EDS analysis was conducted on different PBSA/BC composite granules. As an example, the EDS microanalysis of the PBSA20 cross-section was reported in Figure 10A. Results indicated the presence of inorganic trace elements such as Fe, Na, K, and Ca uniformly distributed within the composite material with concentrations spanning between 0.1 and 0.5 wt.% regardless of the portion examined. These trace elements are typically present within pyrolyzed lignocellulose biomass materials (Figure 10B) and can, therefore, be used to confirm the uniform dispersion of the biochar particles within the PBSA polymer matrix.

## 4. Conclusions

Composites of PBSA and biochar were successfully processed via melt extrusion and injection moulding with different biochar contents (5, 10, 15, and 20 wt.%). Results showed that biochar micro filler (size 5–60 µm) conferred PBSA/BC composites with remarkable mechanical and processability properties. A threshold concentration of 10 wt.% BC was found as the best trade-off between the enhanced mechanical resistance (higher elastic modulus) offered by the filler, and the loss of mechanical plasticity (lower elongation break). The 10 wt.% PBSA/BC composite showed a Young modulus of 0.53 ± 0.06 GPa compared to 0.29 ± 0.05 GPa of the virgin PBSA with a still relatively high stress at break (21.0 ± 0.5 MPa versus 18.0 ± 0.5 MPa) to the detriment of a reduction in the elongation at break (400 ± 53 versus 188 ± 35%). Furthermore, biochar lamellar structure and lignocellulose content had a positive impact on material processability and fluidity reducing polymer viscosity and enhancing melt flow rates at high temperatures. This factor lowered the energy required for polymer extrusion and decreased the occurrences of PBSA polymer chain degradation at high temperatures. Another advantage of biochar was offered by its pigmenting nature, hence the possibility to be used as a renewable filler to replace fuel-based carbon black pigments. In addition, the use of 10 wt.% biochar filler could reduce the overall PBSA/BC composite material cost compared to traditional PBSA/carbon black mixtures. BC is cheaper than carbon black filler which is generally blended with PBSA at much lower concentrations.

In conclusion, biochar filler resulted in a promising candidate as a carbon black substitute to form more sustainable and biodegradable PBSA/BC composites with enhanced processability properties. The use of biochar offers an opportunity for valorising this biomass-derived by-product in agricultural applications such as mulching films, bag liners, and plant pots. To enhance the filmability properties of the PBSA/BC composites and mitigate the stiffness effect given by the biochar, the combined use of biochar and organic plasticizer will be evaluated in a future research study.

## Figures and Tables

**Table 1:** Extrusion temperature profile adopted for the PBSA/BC composites.

Feeder	Zone 1	Zone 2	Zone 3	Head Zone
45 °C	150 °C	165 °C	160 °C	155 °C

**Table 2:** Crystallinity degrees of PBSA and PBSA/BC composites.

Sample	X_C2_ (%)	T_m2_ (°C)	X_C1_ (%)	T_m1_ (°C)	X_c_ (%)
PBSA	0	-	0	87.8	34.4
PBSA5	8.9	78.0	26.3	87.4	35.2
PBSA10	11.2	77.2	23.7	87.2	34.9
PBSA15	13.3	77.1	22.5	87.3	35.8
PBSA20	15.4	76.3	19.3	87.3	34.7

**Table 3:** Mechanical properties of PBSA and PBSA/BC specimens. Mean values ± standard deviation of 5 replicates. (*) Yield strength and elongation at yield equal stress at break and elongation at break, respectively.

Sample	Young Modulus (GPa)	Yield Strength (MPa)	Elongation at Yield (%)	Stress at Break (MPa)	Elongation at Break (%)
PBSA	0.29 ± 0.05	17.5 ± 1.1	14.5 ± 0.3	21.0 ± 0.5	400 ± 53
PBSA5	0.39 ± 0.10	18.9 ± 1.2	13.1 ± 0.4	18.8 ± 0.4	247 ± 66
PBSA10	0.53 ± 0.06	18.6 ± 0.6	12.0 ± 0.9	18.0 ± 0.5	188 ± 35
PBSA15	0.56 ± 0.09	17.6 ± 0.8	9.3 ± 0.8	17.3 ± 0.4	11.6 ± 32
PBSA20	0.71 ± 0.08	*	*	15.3 ± 0.6	5.2 ± 2

## Data Availability

Raw data can be available upon request by contacting the corresponding author. All the processed data are reported in the article text.
